# Emotion Regulation Resources Explain Middle-Aged and Older Adults' COVID-19-Related Distress

**DOI:** 10.1093/geroni/igab046.501

**Published:** 2021-12-17

**Authors:** Cystal Park, Beth Russell, Michael Fendrich

**Affiliations:** 1 University of Connecticut, Storrs Mansfield, Connecticut, United States; 2 UCONN, Storrs Manfield, Connecticut, United States

## Abstract

As the pandemic caused widespread disruption across the world, studies suggested younger adults were faring more poorly than other adults. We hypothesized that younger adults might possess fewer emotion regulation resources and skills, accounting for their greater distress. In a national sample of 1528 adults, we examined how baseline resources (in mid-April, during initial peak infections) predicted distress (depression, anxiety, PTSD symptoms) five weeks later, when states began initial reopenings. Younger adults reported greater distress and less social support, mindfulness, and emotion regulation skills than did middle aged and older adults.. Controlling for stress exposure, younger adults’ distress was predicted by impulsivity and lack of perceived strategies while middle-aged and older adults’ distress was predicted by acceptance of negative emotions; perceived social support was related to lower distress for both groups but mindfulness was unrelated. Results suggest that emotion regulation skills are a promising prevention and intervention focus.

